# Patients with type 2 diabetes inadequately controlled on premixed insulin: effect of initiating insulin glargine plus oral antidiabetic agents on glycaemic control in daily practice*

**DOI:** 10.1111/j.1742-1241.2007.01598.x

**Published:** 2007-12

**Authors:** H Hammer, A Klinge

**Affiliations:** 1 Facharzt für Innere Medizin, Hausärztliche Versorgung Bremen, Germany; 2 Facharzt für Innere Medizin Hamburg, Germany

## Abstract

**Aim:**

Premixed insulin regimens are commonly used for type 2 diabetes mellitus (T2DM) patients. However, there is limited information regarding next-step therapy options in cases where premixed insulin does not provide adequate glycaemic control. This 12-week observational study of everyday clinical practice evaluated the efficacy and safety of insulin glargine (glargine) plus oral antidiabetic drugs (OADs) in T2DM patients previously treated with premixed insulin.

**Methods:**

Type 2 diabetes mellitus patients taking premixed insulin were identified from German clinics and were eligible to switch to glargine plus OADs at the physicians’ and patients’ discretion, as part of routine clinical practice. The study design and conduct was in accordance with German regulations. Fasting blood glucose (FBG), 2-h postprandial blood glucose (PPBG) and glycosylated haemoglobin (HbA_1c_) were measured at the start and after a 12-week observation period.

**Results:**

A total of 5045 patients were followed-up and received glargine plus OADs. FBG [start to end-point: 9.9 ± 2.7 to 6.9 ± 1.5 mmol/l (178 ± 48 to 124 ± 26 mg/dl); p ≤ 0.001], 2-h PPBG [10.8 ± 2.8 to 7.8 ± 1.5 mmol/l (195 ± 50 to 140 ± 27 mg/dl)] and HbA_1c_ (8.3 ± 1.2 to 7.2 ± 0.8%; p ≤ 0.001) improved significantly from start to end-point, respectively. A total of 48.9%, 38.4% and 73.9% of patients had FBG < 6.7 mmol/l (< 120 mg/dl), 2-h PPBG < 7.2 mmol/l (< 130 mg/dl) or HbA_1c_ < 7.5%, respectively, after 12 weeks. Significant reductions in body weight were observed between the start and end of the observation period. A total of 71 adverse events were reported by 38 patients. Hypoglycaemia was the most common event (*n* = 16).

**Conclusions:**

This observational study shows that, in T2DM patients inadequately controlled with premixed insulin, switching therapy to glargine plus OADs is associated with significant improvements in FBG and HbA_1c_, and is well tolerated in everyday clinical practice. Further intensification of insulin therapy, perhaps by adding one or more injections of prandial insulin, would help provide further improvements in glycaemic control in these patients.

**What's known**

Pharmacological therapy of type 2 diabetes mellitus typically starts with oral agents, including metformin or sulfonylurea. However, the natural progression of type 2 diabetes mellitus means that combination therapy is often required. One option to starting insulin therapy is the addition of a ‘basal’ insulin to help manage fasting blood glucose.

**What's new**

Premixed insulin is used by approximately 40% of patients with type 2 diabetes mellitus, but for many people, premixed insulin provides inadequate glycaemic control. In this observational study of everyday clinical practice, transferring from premixed insulin to insulin glargine was associated with significant improvements in HbA_1c_ and fasting blood glucose. Thus, the switch to insulin glargine offers an alternative treatment option for patients with inadequate glycaemic control on premixed insulin.

## Introduction

It is estimated that more than 40% of patients with type 2 diabetes mellitus (T2DM) worldwide use premixed insulin as part of their therapeutic regimen (1). Premixed insulin usually contains a rapid-acting insulin and an intermediate-acting insulin to mimic endogenous insulin secretion patterns and should be taken twice daily, normally before breakfast and dinner (2). While the newer premixed insulin analogues may confer greater improvements in glycaemic control compared with regular human insulin or neutral protamine Hagedorn (NPH) insulin (3), for many patients, premixed insulin alone is insufficient to maintain adequate glycaemic control and is associated with significant day-to-day variability (2).

Optimising fasting blood glucose (FBG) with premixed insulin may, therefore, increase the risk of hypoglycaemia and may not provide sufficient flexibility for patients to achieve optimal glycaemic control (4). The LAPTOP study has demonstrated that, for insulin-naïve patients, a regimen composed of once-daily insulin glargine (LANTUS®; sanofi-aventis, Frankfurt, Germany) plus oral antidiabetic drugs (OADs) is associated with better glycaemic control and reduced risk of hypoglycaemia compared with premixed insulin (4). Despite this, there is limited information regarding therapeutic options for patients for whom premixed insulin provides inadequate glycaemic control or whom frequently experience episodes of hypoglycaemia.

The aim of this observational study was to evaluate the efficacy and safety of insulin glargine with concomitant OADs in everyday clinical practice when used by patients with T2DM who were previously treated with premixed insulin.

## Methods

### Study design

This 12-week study was an open-label, non-interventional, multicentre (*n* = 1791), observational study of patients with T2DM in Germany and based in everyday clinical practice.

This type of study is regulated by the German Drug Law [Arzneimittelgesetz (AMG)] section 67 (6) and is primarily intended to gather knowledge about the safety and efficacy of marketed drugs in daily practice. As part of the sponsor's obligations, the National Association of Statutory Health Insurance Physicians (Kassenärztliche Bundersvereinigungen) was notified of the implementation of this observational study. Owing to the non-interventional nature of this observational study, no ethical approval or informed patient consent was obtained, in accordance with local regulations (AMG), and participation was voluntary. Participating general practitioners (GPs) were asked to document their everyday experience in treating patients with insulin glargine in combination with OADs and received a small compensation for the documentation of each patient, which is common practice for this type of study. All changes in therapy, which were recorded as part of this observational study, were at the discretion of the physician and the patient.

### Patients and study conduct

Patients with T2DM and who were treated with regular human or analogue premixed insulin (most frequently prescribed ratios: 25/75 and 30/70) with or without OADs, were eligible for inclusion in this observational study of everyday clinical practice. The decision to use a therapy regimen including insulin glargine with or without OADs was at the discretion of the physicians and patients, and depended mainly on subjective parameters, as reported by the physician, including: lack of efficacy of premixed insulin, patient wanting a more flexible lifestyle, ability to give up between-meal snacks, frequent occurrence of hypoglycaemia with previous therapy, insufficient mixtures of insulin suspensions available and lack of tolerability with previous therapy. Exclusion criteria were according to the indications and contra-indications (i.e. patients hypersensitive to insulin glargine or any of the excipients) given in the prescribing information and summary of product characteristics for insulin glargine.

At the start of the observation, patients were given insulin glargine to be administered once daily via subcutaneous injection; dosing decisions were at the discretion of the physician, although, when switching from twice-daily NPH insulin, a reduction in the daily dose of insulin by 20–30% is recommended, followed by adjustment of the daily dose (5). Concomitant OAD therapy (dosage, type and changes where necessary) was at the discretion of the physician. All patients in the insulin glargine + OAD set received OADs during the observational study. After the initial visit (week 0; start of observation), subsequent visits were scheduled at weeks 2, 6 and 12 (end of observation).

The case report form specified that up to a maximum of nine blood glucose (BG) values [FBG, 1-h postprandial blood glucose (PPBG) and 2-h PPBG morning, mid-day and evening] should be documented at the start of the observation period and at each follow-up examination. Self-monitored blood glucose (SMBG) was performed using the patients’ own BG meter. Physicians gave all patients training to ensure they could perform SMBG correctly and accurately. Optimum titration of the insulin dose was to the target FBG value of ≤ 5.5 mmol/l (≤ 100 mg/dl) and was measured by the subject via SMBG. An FBG in the range of 5.0–6.7 mmol/l (90–120 mg/dl) and 2-h PPBG in the range of 7.2–8.9 mmol/l (130–160 mg/dl) were considered clinically important to show improvements in glycaemic control with insulin glargine after 12 weeks therapy.

Glycosylated haemoglobin (HbA_1c_) measurements were made at the start and at the end of the observational period using HbA_1c_ measurement systems that were aligned with the original Diabetes Control and Complications Trial method (AlcNow^TM^, Metrika Inc., Sunnyvale, CA, USA or DCA^©^ 2000+ analyzer, Bayer Diagnostics, Eckhart, IN, USA). Owing to the relatively short duration of the observation period, the therapeutic target at week 12 for HbA_1c_ was set at < 7.5%, which was considered clinically important to show improvements in glycaemic control with insulin glargine.

Adverse events (AEs), which included episodes of hypoglycaemia, and adverse drug reactions (ADRs) were reported by the patients either at each visit or as and when they occurred. All events were recorded by the physician. Patients and physicians were not requested to provide details of episodes of hypoglycaemia, such as symptomatic/non-symptomatic or BG levels.

At the end of the observation period, physicians were asked to complete a 5-item questionnaire to investigate how they rated aspects of therapy with insulin glargine (BG control, safety, weight management, patients’ quality of life and demand on physician time). Each item was rated as very good, good, satisfactory or unsatisfactory. Physicians were also asked to note whether therapy with insulin glargine was to be continued. If therapy with insulin glargine was to be discontinued, reasons for discontinuation were to be given.

### Data validation

Dual data entry was performed for some variables (case report form number, patient number, sex and date of birth) to ensure correct identification of patients and prevent duplicate patient entry. Validation methods were used to ensure all data were within plausible ranges (any extreme values were followed up) and in the correct order (e.g. the date listed for the first visit was earlier than the date listed for the last visit; incorrect dates were corrected using the original case report form).

### Population subsets and statistical analysis

All patients who met the inclusion/exclusion criteria were included in the full data set. Five analysis sets were identified for the purpose of this observational study:

- Total population: all patients who were enrolled in this observational study.
- Full data set: patients with T2DM who were previously treated with premixed insulin, with or without OADs prior to the start of observation. Patients in the full data set were treated with insulin glargine with or without OADs during the observation period.
- Prior premixed insulin – OAD set: all patients who were treated with premixed insulin without OADs prior to the observational study. Patients in the prior premixed insulin – OAD group were treated with or without OADs during the observation period.
- Prior premixed insulin + OAD set: all patients who were treated with premixed insulin with OADs prior to the observational study. Patients in the prior premixed insulin + OAD group were treated with or without OADs during the observation period.
- Insulin glargine + OAD set: all patients who were treated with insulin glargine plus OADs during the observational period. Prior to the study, patients in the insulin glargine + OAD set were treated with premixed insulin with or without OADs.

Descriptive statistics are presented. The Wilcoxon signed rank test was used to evaluate changes in FBG, HbA_1c_ and body weight between the start and end of observation. Data are presented as means with standard deviation. Statistical analyses were performed using SPSS for Windows (version 11.01; SPSS Inc., Chicago, IL).

## Results

### Population characteristics at the start of observation

The official start of the observation period was February 2004 and the last available follow up was December 2004. A total of 6560 patients from 1791 centres (general medicine, 56.0%; internal medicine, 27.9%; GP, 13.9%; other/no data available, 2.2%) were included in the analysis of this surveillance study with insulin glargine (safety analysis set). A total of 49.7% were male and 49.7% were female (no data were available for 0.6% of the patients). Median age was 63.2 and 66.3 years, and median body mass index (BMI) was 28.4 and 29.0 kg/m^2^ for males and females, respectively. Characteristics of the subsets of patients at the start of observation are summarised in Table 1.

**Table 1:** Characteristics of patients who were included in this observational study

	Full data set (*n* = 6308)		Prior premixed insulin – OADs (*n* = 3098)		Prior premixed insulin + OADs (*n* = 3210)		Insulin glargine + OAD set (*n* = 5045)	
				
Factor	n	%	n	%	n	%	n	%
Duration of diabetes (years)*	5144	(8.6 ± 6.1)	2497	(8.6 ± 6.3)	2647	(8.7 ± 6.0)	4143	(8.7 ± 5.9)
**Secondary disorders**†								
Patients with disorders	3950	62.6	1890	61.0	2060	64.2	3191	63.3
Micro-albuminuria	2871	45.5	1383	44.6	1488	46.4	2317	45.9
Macro-albuminuria	419	6.6	221	7.1	198	6.2	333	6.6
Retinopathy	1442	22.9	665	21.5	777	24.2	1160	23.0
Neuropathy	2174	34.5	1041	33.6	1133	35.3	1767	35.0
**Prior insulin therapy**‡								
10/90 only	26	0.4	9	0.3	17	0.5	20	0.4
15/85 only	30	0.5	13	0.4	17	0.5	20	0.4
20/80 only	141	2.2	74	2.4	67	2.1	102	2.0
25/75 only	1167	18.5	579	18.7	588	18.3	961	19.1
30/70 only	4381	69.5	2132	68.8	2249	70.1	3509	69.6
40/60 only	72	1.1	35	1.1	37	1.2	45	0.9
50/50 only	315	5.0	163	5.3	152	4.7	250	5.0
Other	34	0.5	11	0.4	23	0.7	27	0.5
More than one formulation	142	2.3	82	2.7	60	1.9	111	2.2
Insulin dose*, †, ‡, §								
25/75 (U/day)	1193	(35.3 ± 15.5)	595	(36.6 ± 14.9)	598	(33.9 ± 16.0)	978	(35.4 ± 15.5)
30/70 (U/day)	4317	(35.2 ± 14.8)	2109	(35.8 ± 14.9)	2208	(34.6 ± 14.6)	3464	(35.3 ± 14.7)
**Prior OAD therapy**†								
Patients taking OADs	3210	50.9	n/a	n/a	3210	100.0	3080	61.1
Glimepiride	847	13.4	n/a	n/a	847	26.4	811	16.1
Glibenclamide	593	9.4	n/a	n/a	593	18.5	559	11.1
Metformin	2192	34.8	n/a	n/a	2192	68.3	2117	42.0
Other OAD¶	160	2.5	n/a	n/a	160	5.0	155	3.1
**Concomitant OAD therapy**								
Patients taking OADs	5045	80.0	1965	63.4	3080	96.0	5045	100.0

As some patients had incomplete data, the numbers of patients with valid data for each characteristic are given. Reasons for missing data were not collected. Results are *n* and per cent except

* mean ± SD.

† Patients can be in more than one subcategory.

‡ Premixed insulin formulation previously used (soluble/protaminated insulin).

§ Mean daily dose of premixed insulin prior to the start of the observation period.

¶ Includes acarbose (full data set, *n* = 61), repaglinide (34), pioglitazone (21), nateglinide (17), rosiglitazone (13) or another OAD (17). Full data set = all patients who fulfilled the inclusion criteria; prior premixed insulin – OAD = all patients who were treated with premixed insulin without OADs prior to the observational study; prior premixed insulin + OAD = all patients who were treated with premixed insulin plus OADs prior to the observational study. Insulin glargine + OAD set = all patients who were treated with insulin glargine plus OADs during the observational period. OADs, oral antidiabetic agents.

Of the 6560 patients that comprised the full data set, 252 patients did not meet the criteria for data analysis (for 244 patients, the previous therapy was not premixed insulin; two patients did not have T2DM; six patients did not meet either criterion). The remaining 6308 T2DM patients were switched from premixed insulin to insulin glargine as part of their clinical management. These patients, thus, comprised the full data set. Of these, a total of 3098 patients were treated with premixed insulin without OADs (prior premixed insulin – OADs) and 3210 patients were treated with premixed insulin plus OADs (prior premixed insulin + OADs), prior to the start of the observation period. A total of 5045 patients were treated with premixed insulin (with or without OADs) prior to the start of the observation period and switched therapy to insulin glargine plus OADs (insulin glargine + OAD set).

At the start of observation, a total of 62.6% of patients in the full data set had experienced one, or more, secondary complications associated with diabetes (Table 1); 45.5%, 34.5%, 22.9% and 6.6% had microalbuminuria, neuropathy, retinopathy or macroalbuminuria, respectively.

In the full data set, the most common reasons given by physicians and patients for switching from premixed insulin to insulin glargine included: lack of efficacy of premixed insulin (68.8%), patient wanting a more flexible lifestyle (55.6%), ability to give up between meal snacks (37.1%) and frequent occurrence of hypoglycaemia with previous therapy (23.5%). Of those citing frequent occurrence of hypoglycaemia (*n* = 1306), the mean number of episodes per person in the 3 months preceding the observation period was 5.0 ± 4.2 (median: 4.0). Less frequent reasons cited for switching from premixed insulin to insulin glargine included insufficient mixtures of insulin suspensions available (13.7%) and lack of tolerability with previous therapy (13.3%).

### Efficacy

**Figure 1:**
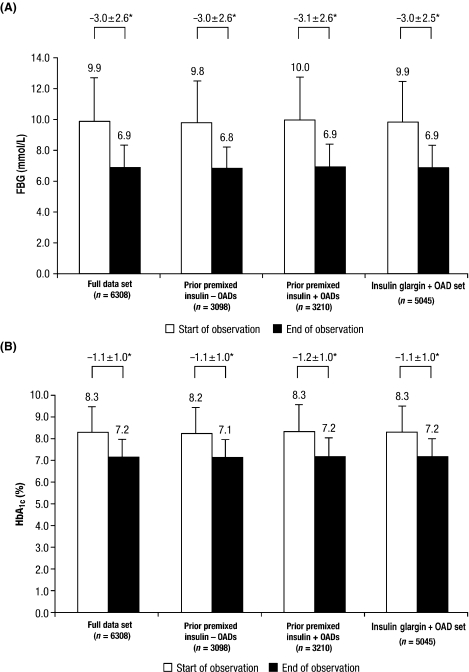
Fasting blood glucose (FBG; A) and glycosylated haemoglobin (HbA_1c_; B) at the start (open bars) and end (closed bars) of the 12-week observation period in the full data set (*n* = 6308; all patients who fulfilled the inclusion criteria), the prior premixed insulin – oral antidiabetic drugs (OADs) set (*n* = 3098; all patients who were treated with premixed insulin without OADs prior to the observational study), the prior premixed insulin + OAD set (*n* = 3210; all patients who were treated with premixed insulin plus OADs prior to the observational study) and the insulin glargine + OAD set (*n* = 5045; all patients who were treated with insulin glargine plus OADs during the observational period). *p ≤ 0.001 for the within-group change in FBG or HbA_1c_ from the start to the end of the observation period

Glycaemic control improved significantly in patients within each subset, with significant improvements in FBG (Figure 1A) and HbA_1c_ (Figure 1B).

In the full data set, the mean decrease in FBG was 3.0 ± 2.6 mmol/l (54.7 ± 46.3 mg/dl) and the median decrease was 2.7 mmol/l [48 mg/dl; interquartile range: −4.2 to −1.5 mmol/l (−75.0 to −27.0 mg/dl)]. As a result, of those patients who provided FBG measurements at the end of the observation (5998 of 6308), 2643 (44.1%) met the therapeutic target for FBG [5.0–6.7 mmol/l (90–120 mg/dl)] and a further 272 (4.5%) patients had FBG < 5.0 mmol/l (< 90 mg/dl) (Table 2). The mean decrease in HbA_1c_ was 1.1 ± 1.0%, with a median of −1.0% (interquartile range: −1.50 to −0.50%). Thus, of 6179 (of 6308) patients for whom HbA_1c_ was measured at the end of the observation, 4593 (74.3%) had HbA_1c_ < 7.5% (Table 2).

**Table 2:** Proportion of patients who achieved clinically relevant fasting blood glucose levels [5.0–6.7 mmol/l (90–120 mg/dl)] or HbA_1c_ (< 7.5%) at week 12

	Full data set (*n* = 6308)		Prior premixed insulin – OADs (*n* = 3098)		Prior premixed insulin + OADs (*n* = 3210)		Insulin glargine + OAD set (*n* = 5045)	
				
	n	%	n	%	n	%	n	%
**FBG**								
Number of patients with FBG measurement	5998		2930		3068		4815	
< 5.0 mmol/l (< 90 mg/dl)	272	4.5	128	4.4	144	4.7	214	4.4
5.0–6.7 mmol/l (90–120 mg/dl)	2643	44.1	1335	45.6	1308	42.6	2139	44.4
Total < 6.7 mmol/l (< 120 mg/dl)	2915	48.6	1463	49.9	1452	47.3	2353	48.9
**HbA**_1c_								
Number of patients with HbA_1c_ measurement	6179		3033		3146		4965	
< 7.5%	4593	74.3	2277	75.1	2316	73.6	3670	73.9

As some patients had incomplete data, the number of patients with valid data for each characteristic are given. Results are *n* and per cent of patients who provided valid data for that characteristic. Reasons for missing data were not collected. Full data set = all patients who fulfilled the inclusion criteria; prior premixed insulin – OAD = all patients who were treated with premixed insulin without OADs prior to the observational study; prior premixed insulin + OAD = all patients who were treated with premixed insulin plus OADs prior to the observational study. Insulin glargine + OAD set = all patients who were treated with insulin glargine plus OADs during the observational period. OAD, oral antidiabetic agent; FBG, fasting blood glucose; HbA_1c_, glycosylated haemoglobin.

Improvements in FBG and HbA_1c_ and the proportions of patients who achieved the target FBG or HbA_1c_ were similar in each of the subsets, including the insulin glargine + OAD set.

In the full data set, reductions in both fasting preprandial BG and 2-h PPBG levels were observed over the 12-week observation period (Table 3) and the majority of patients reached target 2-h PPBG levels of 7.2–8.9 mmol/l (130–160 mg/dl) (Table 3). Similar reductions in both fasting preprandial and PPBG levels were observed in patients who were treated with premixed insulin + OADs or premixed insulin – OADs prior to the observation period (Table 3). In the insulin glargine + OAD set, fasting preprandial and 2-h PPBG levels decreased by 2.9 mmol/l (52 mg/dl) and 3.1 mmol/l (55 mg/dl), respectively (Table 3). A total of 81.6% of patients in the insulin glargine + OAD set achieved the target 2-h PPBG level of 7.2–8.9 mmol/l (130–160 mg/dl).

**Table 3:** Preprandial and 2-h postprandial blood glucose and body weight at the start and at the end of the 12-week observation period

	Full data set (*n* = 6308)		Prior premixed insulin – OADs (*n* = 3098)		Prior premixed insulin + OADs (*n* = 3210)		Insulin glargine + OAD set (*n* = 5045)	
				
Factor	n	Mean ± SD	n	Mean ± SD	n	Mean ± SD	n	Mean ± SD
**Prandial blood glucose**								
*Preprandial*								
Start [mmol/l (mg/dl)]	2870	9.7 ± 2.6 (174 ± 46)	1409	9.6 ± 2.4 (173 ± 44)	1461	9.7 ± 2.7 (175 ± 48)	2265	9.6 ± 2.5 (173 ± 45)
End [mmol/l (mg/dl)]	2366	6.8 ± 1.3 (122 ± 23)	1144	6.7 ± 1.2 (121 ± 22)	1222	6.8 ± 1.3 (122 ± 24)	1896	6.8 ± 1.3 (122 ± 23)
*2-h postprandial*								
Start [mmol/l (mg/dl)]	2870	10.8 ± 2.8 (195 ± 50)	1409	10.8 ± 2.8 (195 ± 50)	1461	10.9 ± 2.8 (196 ± 50)	2265	10.9 ± 2.8 (196 ± 50)
End [mmol/l (mg/dl)]	2366	7.8 ± 1.5 (140 ± 27)	1144	7.8 ± 1.4 (140 ± 25)	1222	7.8 ± 1.6 (140 ± 29)	1896	7.8 ± 1.6 (141 ± 28)
*Proportion of patients reaching postprandial therapeutic target*								
Number of patients with valid data	2183		1075		1108		1719	
< 7.2 mmol/l (< 130 mg/dl)*	839	38.4	399	37.1	440	39.7	639	37.2
7.2–8.9 mmol/l (130–160 mg/dl)*	966	44.3	493	45.9	473	42.7	763	44.4
Total < 8.9 mmol/l (< 160 mg/dl)*	1805	82.7	892	83.0	913	82.4	1402	81.6
**Body weight**								
Start (kg)	6175	84.8 ± 14.4	3030	84.0 ± 14.2	3145	85.6 ± 14.6	4958	85.2 ± 14.4
End (kg)	6175	83.3 ± 14.1	3030	82.5 ± 14.0	3145	84.0 ± 14.2	4958	83.6 ± 14.1
Change		−1.5 ± 3.3†		−1.5 ± 3.3†		−1.6 ± 3.3†		−1.5 ± 3.2†

As some patients had incomplete data, the numbers of patients with valid data for each characteristic are given. Reasons for missing data were not collected. Results are means ± SD

* *n*, per cent

† p ≤ 0.001 for within-group change in body weight from start of observation. Full data set = all patients who fulfilled the inclusion criteria; prior premixed insulin – OAD = all patients who were treated with premixed insulin without OADs prior to the observational study; Prior premixed insulin + OAD = all patients who were treated with premixed insulin plus OADs prior to the observational study. Insulin glargine + OAD set = all patients who were treated with insulin glargine plus OADs during the observational period. SD, standard deviation; OAD, oral antidiabetic agents.

### Body weight

In the full data set, significant reductions in body weight were observed between the start and end of the observation period (−1.5 ± 3.3 kg; p ≤ 0.001; median change in body weight: −1.0 kg; interquartile range: −3 to 0 kg) (Table 3). Reductions in body weight were of a similar magnitude and were statistically significant in the other subsets of the study population.

### Insulin dose and oral antidiabetic therapy

In the full data set, the mean daily dose of insulin increased by 4 U between week 0 (22.1 ± 10.6 U) and week 12 (26.3 ± 11.6 U). A similarly low increase in the daily insulin dose was seen in patients in the other subsets of the total population.

In the insulin glargine + OAD group, there were no changes in the frequency of patients taking each OAD between the start and end of the observation; metformin (54.8% vs. 58.2% for start vs. end of observation) glimepiride (40.5% vs. 44.5%) and glibenclamide (10.9% vs. 9.8%) were used most frequently. The proportion of patients taking other OADs was 4.4% and 5.2% at the start and end of observation, respectively.

### Safety

All patients (*n* = 6560) were included in the full data set. A total of 71 AEs were reported in 38 patients; of these, 33 events (17 patients) were classed as serious AEs. Hypoglycaemia was the most commonly reported AE (16 events), followed by hyperhydrosis (three events).

A total of 30 events in 14 patients were classed as ADRs; hypoglycaemia was the most commonly reported ADR (13 events), followed by hyperhydrosis (three events), over the 12-week observation period. Two patients died during the course of the observation period; one was due to acute kidney failure, which was not considered related to the treatment regimen, but no indication was available for the second, thus it was not possible to determine whether it was related to treatment or not.

### Physician assessment of insulin glargine therapy

In the full data set, physicians consistently rated insulin glargine therapy as very good or good for the rated aspects of therapy (Table 4) – quality of life (94.1%), BG control (87.6%) and weight management (68.9%). Only 2.9% of physicians rated the treatment safety of insulin glargine as satisfactory or unsatisfactory, and 95.9% rated treatment safety with insulin glargine as good or very good. Physicians also reported that the demands placed on their time with insulin glargine were either very good or good (89.7%).

**Table 4:** Physician assessment of insulin glargine therapy

	Full data set (*n* = 6308)		Prior premixed insulin – OADs (*n* = 3098)		Prior premixed insulin + OADs (*n* = 3210)		Insulin glargine + OAD set (*n* = 5045)	
				
Factor	n	%	n	%	n	%	n	%
**Quality of life**								
Very good	2615	41.5	1308	42.2	1307	40.7	2085	41.3
Good	3320	52.6	1626	52.5	1694	52.8	2673	53.0
Satisfactory	277	4.4	118	3.8	159	5.0	221	4.4
Unsatisfactory	19	0.3	7	0.2	12	0.4	15	0.3
No response given	77	1.2	39	1.3	38	1.2	51	1.0
**Blood glucose control**								
Very good	2921	46.3	1479	47.7	1442	44.9	2310	45.8
Good	2606	41.3	1259	40.6	1347	42.0	2103	41.7
Satisfactory	602	9.5	274	8.8	328	10.2	491	9.7
Unsatisfactory	118	1.9	58	1.9	60	1.9	101	2.0
No response given	61	1.0	28	0.9	33	1.0	40	0.8
**Weight management**								
Very good	1691	26.8	863	27.9	828	25.8	1335	26.5
Good	2655	42.1	1335	43.1	1320	41.1	2136	42.3
Satisfactory	1455	23.1	675	21.8	780	24.3	1182	23.4
Unsatisfactory	422	6.7	183	5.9	239	7.5	334	6.6
No response given	85	1.4	42	1.4	43	1.3	58	1.2
**Safety**								
Very good	3426	54.3	1683	54.3	1743	54.3	2765	54.8
Good	2625	41.6	1280	41.3	1345	41.9	2093	41.5
Satisfactory	164	2.6	92	3.0	72	2.2	123	2.4
Unsatisfactory	19	0.3	8	0.3	11	0.3	15	0.3
No response given	74	1.2	35	1.1	39	1.2	49	1.0
**Demand on time**								
Very good	2051	32.5	1036	33.4	1015	31.6	1592	31.6
Good	3608	57.2	1744	56.3	1864	58.1	2959	58.7
Satisfactory	528	8.4	258	8.3	270	8.4	408	8.1
Unsatisfactory	32	0.5	13	0.4	19	0.6	25	0.5
No response given	89	1.4	47	1.5	42	1.3	61	1.2

As some patients had incomplete data the numbers of patients with valid data for each characteristic are given. Reasons for missing data were not collected. Results are *n* and per cent. Full data set = all patients who fulfilled the inclusion criteria; prior premixed insulin – OAD = all patients who were treated with premixed insulin without OADs prior to the observational study; prior premixed insulin + OAD = all patients who were treated with premixed insulin plus OADs prior to the observational study. Insulin glargine + OAD set = all patients who were treated with insulin glargine plus OADs during the observational period. OADs, oral antidiabetic agents.

In the full data set, continuation of insulin glargine therapy was planned for 6163 patients (97.7%) at the end of the observation period. Of the 116 patients who stopped therapy with insulin glargine, the most common reasons were ‘inadequate BG control’ (*n* = 53), ‘change of therapy’ (*n* = 42), ‘patient lost to follow-up’ (*n* = 28) or ‘treatment no longer considered necessary at the end of observation’ (*n* = 5). For 29 patients, there was no information on whether insulin glargine treatment was continued at the end of the observation period. In the insulin glargine + OAD set, continuation of insulin glargine plus OAD therapy was planned for 4929 patients (97.7%).

## Discussion

Here, we describe a non-interventional, non-randomised observational study that was undertaken to document postmarketing experience of transferring patients with T2DM from premixed insulin to insulin glargine. This study demonstrates for the first time in daily clinical practice that initiation of insulin glargine with or without OADs improves glycaemic control in patients with T2DM who were poorly controlled with premixed insulin prior to the observation period. Significant improvements in both HbA_1c_ and FBG were observed during the 12-week observation period in patients treated with insulin glargine with or without OADs. Furthermore, body weight was reduced over the 12-week observational period with relatively small increases in daily insulin dose. The improvements in FBG, HbA_1c_ and body weight were also observed in the subsets of the full data population, including the insulin glargine + OAD set.

The results presented in this observational study were broadly consistent, irrespective of the patient subsets, and show that the initiation of insulin glargine in patients previously treated with premixed insulin was associated with significant improvements in FBG, HbA_1c_ and body weight, irrespective of whether or not patients were previously taking OADs.

In a recent observational study of 12,216 patients with T2DM poorly controlled with OADs alone [HbA_1c_: 8.7 ± 1.4%; FBG: 11.2 ± 3.1 mmol/l (202 ± 56 mg/dl)], addition of once-daily insulin glargine was associated with improvements in FBG [7.4 ± 1.8 mmol/l (133 ± 33 mg/dl)] and HbA_1c_ (7.2 ± 0.9%) at 3 months, which were maintained at 9 months (6). In our study, we show that switching from premixed insulin to insulin glargine is associated with significant improvements in FBG and HbA_1c_ after only 12 weeks. Furthermore, the majority of patients achieved the target FBG, HbA_1c_ or PPBG.

Premixed insulin is a common therapy for T2DM and approximately 40% of patients are treated with premixed insulin worldwide (1), while a recent German study suggested that premixed insulin constitutes the majority (> 80%) of insulin usage in patients with either T1 or T2DM (7).

In the transition to insulin glargine therapy, OADs are typically used in combination. In the present study, some of the patients previously treated with premixed insulin (with or without oral antidiabetic agents) switched to insulin glargine, but did not take concomitant oral therapy. In those who switched from premixed insulin without or with OADs to insulin glargine without OADs, improvements in HbA_1c_, FBG and PPBG were consistent with the insulin glargine + OAD and the full data sets (data not shown).

Weight gain is a seemingly unavoidable occurrence with initiation of insulin therapy in T2DM (8). In a 28-week study comparing initiation of insulin therapy with either biphasic insulin aspart (70/30) or insulin glargine, once-daily insulin glargine was associated with significantly less weight gain compared with twice-daily biphasic insulin aspart (+3.5 vs. +5.4 kg; p < 0.01) (9). Furthermore, educational programmes, if combined with insulin glargine therapy, may help prevent the weight gain otherwise associated with insulin therapy (10). In this study, the authors used a programme comprising 90-min lessons over a 12-week duration, with focus on various topics, such as ‘insulin dosing and injection’, ‘BG self-monitoring’, ‘food and diet’, ‘physical exercise’ and ‘diabetic complications’. Patients were monitored for 30 months and were not to make dietary adjustments as part of the study. No weight gain was seen in the overall study population. In our study, we show that weight loss can be achieved in clinical practice (mean weight change: −1.5 ± 3.3 kg; median: −1.0 kg; interquartile range: −3.0 to 0.0 kg), and that this occurs in conjunction with the majority of patients reaching target FBG, HbA_1c_ (74.3% of patients with HbA_1c_ < 7.5%) or PPBG [82.7% of patients with PPBG < 8.9 mmol/l (< 160 mg/dl)]. This is in support of a previous observational study of everyday clinical practice, where insulin glargine was initiated for 12,216 patients with T2DM previously inadequately controlled by OAD therapy (6), where BMI decreased by 0.3 kg/m^2^ at 3 months (*n* = 10,692), from 29.0 ± 4.7 kg/m^2^ at the start of observation. At the 9-month follow up, mean change in BMI from the start of observation was −0.5 kg/m^2^ (*n* = 5324), and was greatest (−2.1 kg/m^2^) in those patients who had BMI ≥ 35 kg/m^2^ at the start of observation. The lower dose of external insulin glargine plus the mobilisation of endogenous insulin by OADs compared with the higher dose of external premixed insulin at the start of the observation may be associated with the weight loss observed in our study.

A further barrier to the initiation of insulin therapy for T2DM is hypoglycaemia (11). In a 24-week randomised controlled trial (RCT) comparing initiation of basal insulin added to OADs vs. twice-daily premixed insulin, the majority of patients in both groups (61.6% vs. 67.2%) experienced at least one episode of hypoglycaemia, but the prevalence was significantly higher in the premixed insulin group (4). In our study, only 16 patients reported an episode of hypoglycaemia, of which 13 were classed as ADRs. However, this may partly be due to the less stringent target levels used in everyday clinical practice compared with the study by Janka et al. (4), which set target values of HbA_1c_ ≤ 7.0% and FBG ≤ 5.6 mmol/l (≤ 100 mg/dl).

In a 28-week RCT comparing insulin glargine with NPH insulin in patients who were inadequately controlled (HbA_1c_ 7.0–12.0%) with insulin, < 30% of patients achieved target FBG levels [< 6.7 mmol/l (< 120 mg/dl)] at the study end (12). The authors reported that this may have been due to reluctance of the investigators and/or subjects to intensively titrate the insulin dose likely because of fear of hypoglycaemia or weight gain. In our study, almost 50% of patients achieved FBG levels of ≤ 6.7 mmol/l (≤ 120 mg/dl) and > 70% achieved HbA_1c_ levels of ≤ 7.5%.

There are limitations of the study that mean care should be taken when interpreting our results. First, as this was an observational study and not controlled by the inclusion of a comparator arm, it is likely that some of the improvements observed may be related to a study effect rather than the therapeutic regimen based on insulin glargine plus OADs. Unfortunately, as this study was uncontrolled, it is not possible to delineate between the study effect and the regimen used. Nevertheless, the improvements in glycaemic control over the study period are consistent with RCTs (13,14).

Second, the duration of the observation period was restricted to 12 weeks; however, subsequent follow ups are planned at 1 year to address longer-term benefits of insulin glargine plus OADs in clinical practice, particularly on the overall weight loss and low incidence of hypoglycaemia observed here.

In the present study, the target HbA_1c_ was ≤ 7.5% and was achieved by almost 75% of the patients. However, the International Diabetes Federation (15) and the American Diabetes Association (16) have set target HbA_1c_ levels of ≤ 6.5% and ≤ 7.0%, respectively. It is likely that if the target HbA_1c_ had been set at equivalent levels, a more intensive treatment algorithm would be needed, which may, however, increase risk of hypoglycaemia. Thus, the low incidence of hypoglycaemia observed in the present study may reflect the less stringent titration targets used [i.e. FBG < 6.7 mmol/l (< 120 mg/dl) and HbA_1c_ < 7.5%]. However, a number of trials have demonstrated that greater improvements in FBG and/or HbA_1c_ can be achieved with insulin glargine vs. NPH insulin, with a lower risk of hypoglycaemia, even with intensive insulin titration regimens (13,14,17). The incidence of hypoglycaemia may also reflect the self-report methods used, as patients and physicians were not requested to provide specific details of each episode. Thus, the occurrence of hypoglycaemia may have been underreported by patients in the present study.

In the present study, approximately 75% of patients met our target HbA_1c_ level of ≤ 7.5%, with a mean of 7.2% for the full data set. While this is close to the ADA target (and undoubtedly, some patients will have met the ADA, and possibly IDF targets), we suggest that further intensification of therapy, perhaps by adding one or more bolus insulin injections, would help improve glycaemic control further (18). Indeed, the improvements observed in the present study provide a foundation on which further therapies should be added.

In summary, in this observational study of everyday clinical practice, we have shown here for the first time that once daily insulin glargine plus OADs is an effective therapeutic regimen with a good safety profile for patients with T2DM who were inadequately controlled with premixed insulin. Furthermore, in a clinical setting, improvements in glycaemic control can be achieved with low risk of weight gain and with a low prevalence of hypoglycaemia in a large study population (6308 patients). It remains to be seen whether these improvements are maintained after a longer duration of time in this cohort of patients.
